# Supplementary figures and images for: Sclerostin inhibits interleukin-1β-induced late stage chondrogenic differentiation through downregulation of Wnt/β-catenin signaling pathway
Source: PLoS One. 2020 Sep 25;15(9):e0239651. doi: 10.1371/journal.pone.0239651 (PMC7518574; doi:10.1371/journal.pone.0239651)

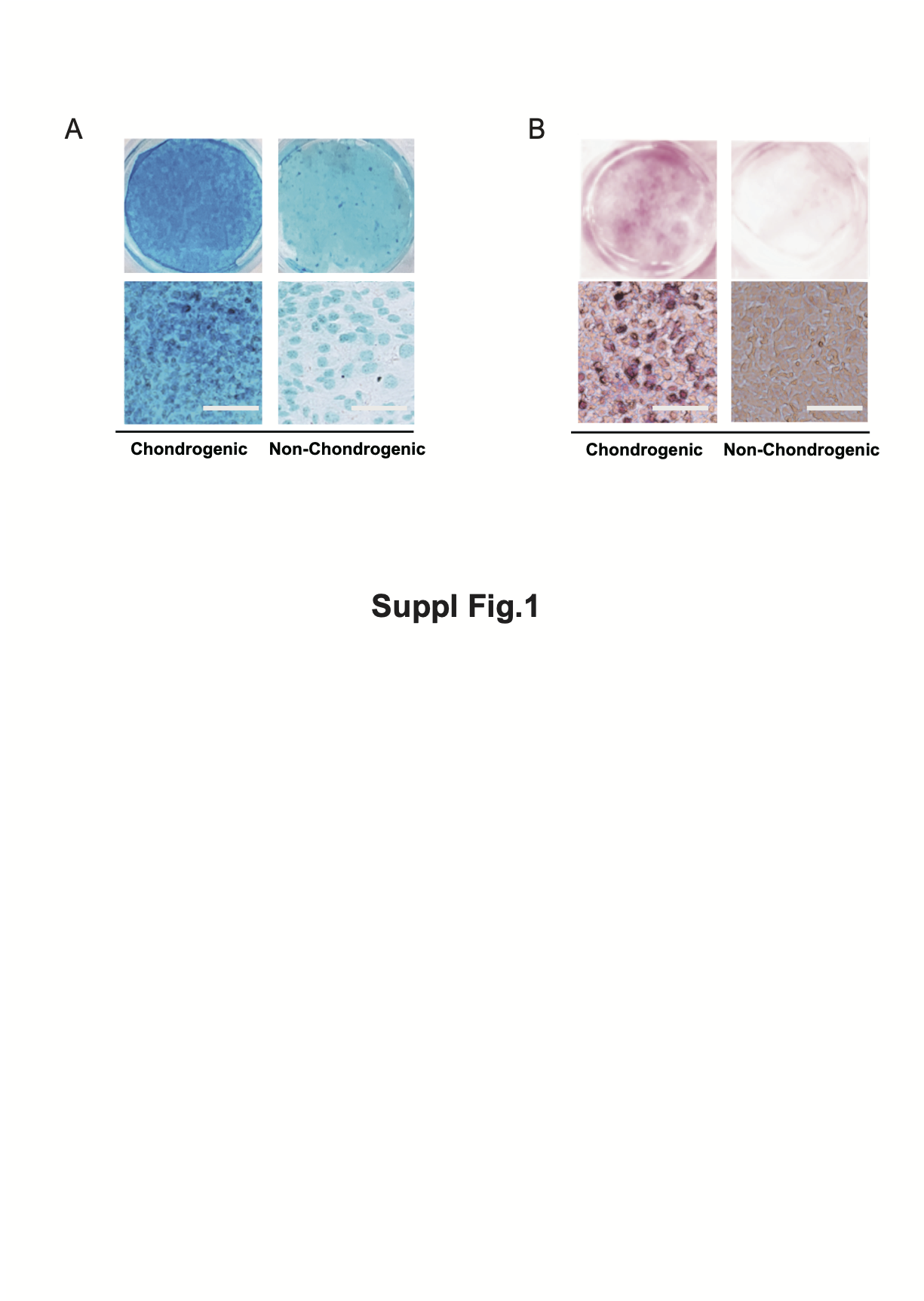

Supplement: S1 Fig — (A) Alcian Blue staining of ATDC5 cells at 3 weeks of culture in chondrogenic media (left) and non-chondrogenic media (right). Less intense staining is observed in non-chondrogenic media. Scale bars = 100 μm. (B) Alizarin red staining of ATDC5 cells at 7 weeks of culture in chondrogenic media (left) and non-chondrogenic media (right). Less intense staining is observed in non-chondrogenic media. Scale bars = 100 μm. (TIFF) [file pone.0239651.s001.tiff]

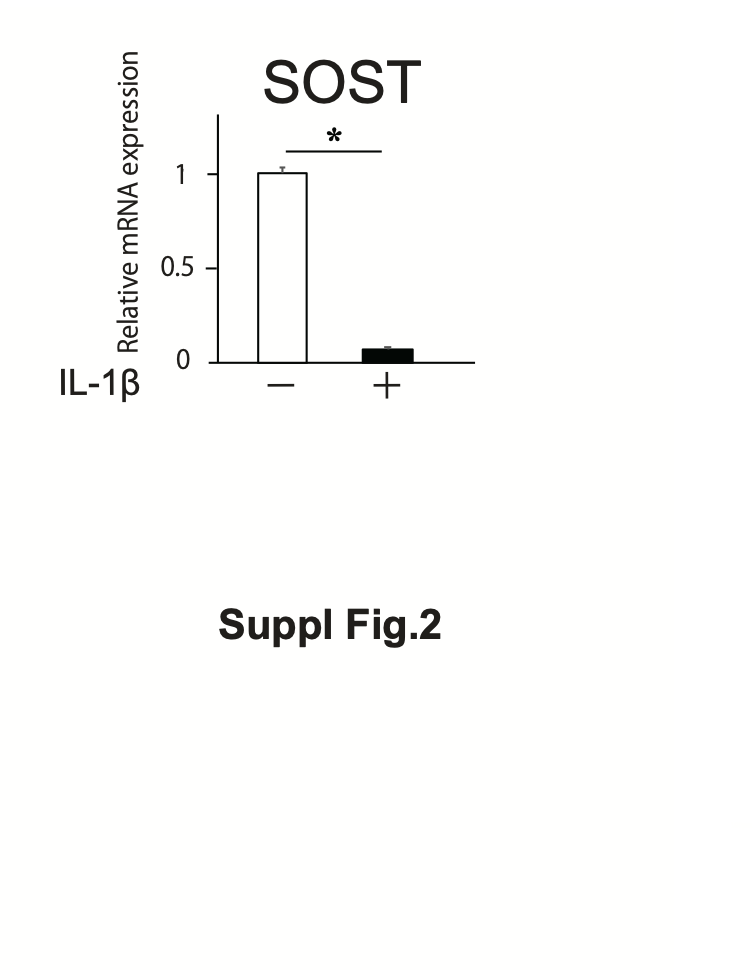

Supplement: S2 Fig — The relative mRNA expression of SOST is significantly decreased in the cells with addition of IL-1β. N = 4 *P<0.05. (TIFF) [file pone.0239651.s002.tiff]
