# Supplementary material for: Sclerostin inhibits interleukin-1β-induced late stage chondrogenic differentiation through downregulation of Wnt/β-catenin signaling pathway
Source: PLoS One. 2020 Sep 25;15(9):e0239651. doi: 10.1371/journal.pone.0239651 (PMC7518574; doi:10.1371/journal.pone.0239651)
Supplement: Attachment — Submitted filename: response to reviewers_200721.docx [file pone.0239651.s003.docx]

**Response to Reviewers**

Please find in the following paragraphs a point-by-point reply to the comments of the reviewers (*in italics*).

Journal Requirements:

*When submitting your revision, we need you to address these additional requirements.*

*1. Please ensure that your manuscript meets PLOS ONE's style requirements, including those for file naming. The PLOS ONE style templates can be found at*

*http://www.journals.plos.org/plosone/s/file?id=wjVg/PLOSOne_formatting_sample_main_body.pdf and http://www.journals.plos.org/plosone/s/file?id=ba62/PLOSOne_formatting_sample_title_authors_affiliations.pdf*

**Response:** The manuscript style was checked according to above templates.

*2. We noticed you have some minor occurrence of overlapping text with the following previous publication(s), which needs to be addressed:*

*https://journals.plos.org/plosone/article?id=10.1371%2Fjournal.pone.0201839*

*We appreciate that the above is your own previously published work, but do not feel that text reuse outside of the Material and Methods is best practice and we would ask that you rephrase these sections. In addition as part of your revision we would recommend that you clearly indicate where methods are based on methods previously described in previous publication (e.g. As previously described [Reference],...)*

*Further consideration is dependent on these concerns being addressed.*

**Response:** In accordance with the request, some of the sentences indicated above were deleted or revised, and the description “As described in detail previously [Reference],…” was added appropriately.

*3. Thank you for stating the following in the Acknowledgments Section of your manuscript:*

*"This work was supported in part by a Grant-in-Aid for Scientific Research from*

*the Ministry of Education, Culture, Sports, Science and Technology of Japan (#25462347)."*

*We note that you have provided funding information that is not currently declared in your Funding Statement. However, funding information should not appear in the Acknowledgments section or other areas of your manuscript. We will only publish funding information present in the Funding Statement section of the online submission form.*

*Please remove any funding-related text from the manuscript and let us know how you would like to update your Funding Statement. Currently, your Funding Statement reads as follows:*

*The authors received no specific funding for this work.*

**Response:** We apologize this incorrect writing. We should revise funding information in the Funding Statement section of the online submission form.

Review Comments to the Author

**Reviewer #1:** *In this manuscript, the authors use the ATDC5 cell line to examine the dual effects of sclerostin and interleukin 1 beta on cell differentiation, cell mineralisation and gene expression of chondrocyte markers and components of the wnt signalling pathway. They found that IL-1B impaired chondrocyte differentiation, and that the addition of sclerostin had no additional effect on this. IL-1B also drove chondrocyte mineralisation and sclerostin was able to restore this.*

*1) I am unclear as to the novelty of this work, as the dual effects of sclerostin and inflammatory cyotkines have been previously investigated (e.g. doi: 10.1016/j.joca.2011.04.014; doi: 10.3892/mmr.2017.6278; doi: 10.1186/s13075-015-0540-6).*

**Response:** The strong point of this study is the investigation of these effects through whole process of chondrogenic differentiation from mesenchymal chondroprogenitor to terminal calcification. Especially, the role of inflammatory cyotkines and sclerostin in the terminal calcification of chondrocyte has not been investigated in the previous studies, which it is considered as the novelty of the present study. Additional comments were described in the discussion (Line 186-198).

*2) I have concerns with some of the data - in particular the alizarin red staining (and alcian blue) which appear to have staining outside of the well and therefore make me question these results. Higher power is also needed to confirm this is not ectopic calcification. Further, some of the stats are questionable - e.g. Fig 3B VEGF has huge error bars which are significant?*

**Response:** The images of alizarin red and alcian blue were replaced with new ones. Photomicrographs with high power magnification were added to the data. Relative mRNA expression of VEGF was re-analyzed by real-time PCR, and the data were replaced in Fig. 3B.

*3) Some of the statements in the results are incorrect e.g. line 118 - only one marker was significantly changed and this statement suggests all were. This needs rectifying throughout*

**Response:** We apologize this incorrect writing. The description ‘The mRNA expressions of markers for cartilage catabolism were’ was revised to ‘The mRNA expression of MMP-13, one of markers for cartilage catabolism, was’ (Line 142-143).

*4) Were other markers of endochondral ossificaion examined - e.g. PTH/PTHrP/Ihh? The authors looked at ADAMTS5, but what about TIMPs?*

**Response:** Unfortunately, the other markers including PTH/PTHrP/Ihh and TIMPs were not investigated. We would like to analyze them in the future opportunity.

*5) Justification of concentrations used required*

**Response:** IL-1β was not cytotoxic up to a concentration of 100 ng/mL (Graeser et al. 2009). Of these concentrations, 10 ng/mL IL-1β is considered as an optimized concentration to induce OA-like condition in vitro, and this concentration was also used in most of previous studies (Shi et al. 2016, Wang et al. 2019). Text was revised (Line 217- 219) and following references were added.

12. Graeser AC, et al. Synergistic chondroprotective effect of alpha-tocopherol, ascorbic acid, and selenium as well as glucosamine and chondroitin on oxidant induced cell death and inhibition of matrix metalloproteinase-3--studies in cultured chondrocytes. Molecules. 2009; 15: 27-39.

13. Shi S, et al. Silencing of Wnt5a prevents interleukin-1beta-induced collagen type II degradation in rat chondrocytes. Exp Ther Med. 2016; 12: 3161-3166.

14. Wang F, et al. IL-1beta receptor antagonist (IL-1Ra) combined with autophagy inducer (TAT-Beclin1) is an effective alternative for attenuating extracellular matrix degradation in rat and human osteoarthritis chondrocytes. Arthritis Res Ther. 2019; 21: 171.

**Reviewer #2:** *In this work, Miyatake and colleagues provide a straightforward study showing the potential of sclerostin to inhibit canonical Wnt signaling and be a therapeutic target for osteoarthritis (OA). This study provides incremental knowledge of sclerostin’s role in an IL1-β induced OA in vitro model. I am not convinced that the cell line used in this study is the best model to look at early and later stages of chondrogenic differentiation in the context of endochondral ossification, especially as these are teratoma-derived if I understand correctly. The authors need to speak to why this cell line was used.*

**Response:** The following description was added to the revised manuscript (Line 186-198).

As described in detail previously [15, 16], ATDC5 is a good model system for studying the dynamic processes of chondrogenesis, and many findings in this system may have relevance to chondrogenesis in vivo. In our previous study, the role of sclerostin as an inhibitor of the canonical Wnt signaling pathway in the chondrogenic differentiation could be characterized using the same model system [11]. This established model system was used to investigate the effects on chondrogenic differentiation at the different timing of early and later stages in the present study. Although several in vivo OA models have been developed to investigate the pathological feature and therapeutic effects, they need to be considered influence under the multifactorial and complex conditions including mechanical load, synovial inflammation, cartilage degeneration and abnormal bone remodeling. This study simply focused the effect of sclerostin on the cytokine among several factors associated with OA pathogenesis.

*Below are additional comments to the authors:*

*Introduction*

*- Would be beneficial to reference what is known from in vivo models of sclerostin and canonical Wnt pathway components, specifically knock-out or transgenic mouse models, to show physiological, whole system effects.*

**Response:** Descriptions about in vivo models of sclerostin and canonical Wnt pathway components were added to the introduction section (Line 33-35, 40-43).

*Methods*

*- Need to provide more detail about the source/company, species, and phenotype of the ATDC5 cells.*

**Response:** Accordingly, description about ATDC5 was added to the text (Line 58-60).

*- Concerned about cells cultured through 7 weeks in 2D and the environment affecting phenotype more than the pharmacological treatment*

**Response:** Since it is difficult to create the model for terminal calcification in 3D culture of chondrocyte, we used 2D culture model. Addition of IL-1β accelerated this phenotypic change, but treatment with sclerostin showed significant effects of restoration even in this environment.

*- Line 63, clarify that for early stage treatment was started at 24 hours after confluency and for late stage, treatment was started on day 21 as the wording right in this current draft is confusing.*

**Response:** The description was revised as suggested (Line 73-75).

*- Why standard error of the mean vs standard deviation for plots?*

**Response:** It was our mistake. All data were given as mean and standard deviation. The statement was revised (Line 130).

*- Need to explain and/or reference why 10 ng/mL IL-1β was used to induce inflammation vs. other concentrations.*

**Response:** IL-1β was not cytotoxic up to a concentration of 100 ng/mL (Graeser et al. 2009). Of these concentrations, 10 ng/mL IL-1β is considered as an optimized concentration to induce OA-like condition in vitro, and this concentration was also used in most of previous studies (Shi et al. 2016, Wang et al. 2019). Text was revised and following references were added (Line 217- 219).

12. Graeser AC, et al. Synergistic chondroprotective effect of alpha-tocopherol, ascorbic acid, and selenium as well as glucosamine and chondroitin on oxidant induced cell death and inhibition of matrix metalloproteinase-3--studies in cultured chondrocytes. Molecules. 2009; 15: 27-39.

13. Shi S, et al. Silencing of Wnt5a prevents interleukin-1beta-induced collagen type II degradation in rat chondrocytes. Exp Ther Med. 2016; 12: 3161-3166.

14. Wang F, et al. IL-1beta receptor antagonist (IL-1Ra) combined with autophagy inducer (TAT-Beclin1) is an effective alternative for attenuating extracellular matrix degradation in rat and human osteoarthritis chondrocytes. Arthritis Res Ther. 2019; 21: 171.

*- Need a control group of cells that have not been subjected to chondrogenic media to characterize and compare how these cells behave over the treatment period in 2D culture.*

**Response:** To characterize the cells that are not subjected to chondrogenic media, ATDC5 cells were also cultured without ITS. Chondrogenic differentiation with proteoglycan synthesis was confirmed by positive staining with Alcian blue after 3 weeks (Supple Fig. 1a). Less intense staining was observed without ITS. Terminal calcification was confirmed by positive staining with Alizarin red after 7 weeks (Supple Fig. 1b). Less staining was identified without ITS.

*- Would be helpful to look at protein levels (immunostaining if not westerns) of active beta-catenin and downstream targets (e.g. Axin 1/2) to further confirm sclerostin’s effects.*

**Response:** Accordingly, immunostaining for ß-catenin, Axin1, and Axin 2 was performed and data were added (Fig. 2 and Fig. 4).

*Discussion*

*- Would be beneficial to provide commentary on how this knowledge could be used for treatment in diseases such as osteoarthritis, in context with this in vitro model compared to in vivo models.*

**Response:** Following comment was added to the revised text (Line 193-198).

Although several in vivo OA models have been developed to investigate the pathological feature and therapeutic effects, they need to be considered influence under the multifactorial and complex conditions including mechanical load, synovial inflammation, cartilage degeneration and abnormal bone remodeling. This study simply focused the effect of sclerostin on the cytokine among several factors associated with OA pathogenesis.

**Reviewer #3:** *This manuscript reports that sclerostin prevents late stage of chondrogenic differentiation induced by interleukin-1ß in ATDC5 cells through inhibition of Wnt/ß-catenin signaling. The authors show that sclerostin has no effect on the expression of chondrogenic differentiation markers and Wnt/ß-catenin pathway genes in response to interleukin-1ß at early stage (Figs 1 and 2). However, sclerostin counteracts the effect of interleukin-1ß on the expression of most these genes at late stage (Figs 3 and 4). Although this study indicates an antagonistic effect of sclerostin on interleukin-1ß in chondrogenic differentiation, the present results are too preliminary to warrant a publication.*

*1. Interleukin-1ß treatment increases the expression of some Wnt/ß-catenin genes, and inhibits the expression of others (for example, axin2), but the extent of increase or decrease is very limited for most genes. What would be the overall outcome of these regulations on Wnt/ß-catenin signaling? It is necessary to assess whether Wnt/signaling is indeed increased in the cells treated interleukin-1ß. Similarly, the authors should also examine whether sclerostin exerts a net effect on Wnt/signaling in cells treated by interleukin-1ß.*

**Response:** As reviewer suggested, effects of interleukin-1ß on LRP5/6 seems to be limited. However, expression of phosphorylated LRP6 was increased by interleukin-1ß (Fig. 4b). Gene expression of Axin2 was re-examined and data showed the same trend as the other related genes. We apologize the data in the initial submission were not correct due to technical error and the graph was replaced in the revised figure. In addition, we assessed the expression of SOST in the cells during chondeogenic differentiation with or without Interleukin-1ß (Supple Fig. 2). Interleukin-1ß significantly suppressed the expression of SOST. This is thought to affect indirectly upregulation of Wn/ß-catenin signaling. Similar mechanism was reported by Yoshida et al (Inflammation 2018, [31]). Interleukin-1ß activated Wnt/ß-catenin signaling by suppression of Wnt antagonist, DKK-1.

*2. It is not clear how sclerostin regulates the expression of these genes in the absence of interleukin-1ß. It inhibits the expression of Wnt/ß-catenin pathway genes directly or indirectly by inhibiting bone formation? Sclerostin is a secreted protein that antagonizes Wnt signaling by binding to LRP5/6, how it inhibits axin1 and ctnb-1 (ß-catenin) expression, is there a positive feedback of Wnt/ß-catenin signaling in the cells at this stage of chondrogenic differentiation.*

**Response:** Our previous study (Yamaguchi et al. PLOS ONE 2018 [10]) demonstrated that sclerostin directly inhibited the expression of Wnt/ß-catenin pathway genes with downregulation of LRP5/6 during chondrogenic differentiation in the absence of interleukin-1ß. When added to the interleukin-1ß, sclerostin suppressed the expressions of LRP6 and phosphorylated LRP6 in the present study. It is unclear whether there is a positive feedback of Wnt/ß-catenin signaling in the cells.

*3. The effect of sclerostin on BMP signaling needs to be examined to see if it inhibits chondrogenic differentiation also by antagonizing this pathway.*

**Response:** The mRNA expression level of BMP-2 was additionally investigated and added to ta data (Fig 3c). BMP2 was upregulated by interleukin-1ß, and it was inhibited by sclerostin.
