# Supplementary material for: Sclerostin inhibits interleukin-1β-induced late stage chondrogenic differentiation through downregulation of Wnt/β-catenin signaling pathway
Source: PLoS One. 2020 Sep 25;15(9):e0239651. doi: 10.1371/journal.pone.0239651 (PMC7518574; doi:10.1371/journal.pone.0239651)
Supplement: Attachment — Submitted filename: response to reviewers_rev2.docx [file pone.0239651.s004.docx]

**Response to Reviewers**

Please find in the following paragraphs a point-by-point reply to the comments of the reviewers (*in italics*).

***Reviewer #1:*** *(No Response)*

***Reviewer #2:*** *The authors have addressed all of the original comments and critiques except the issue with standard deviation vs. standard error of the mean. In the comments to reviewer, the authors comment that standard deviation was calculated and used to show variance in the data sets. However, the text reads "standard error" which is not standard deviation - as it is the standard deviation divided by the square root of the sample size. Please correct accurately and update in the text.*

**Response:** We apologize incorrect description. In the text, "standard error" was corrected to "standard deviation" (Line 130).

***Reviewer #3:*** *This revised manuscript includes additional experiments examining the effects of IL-1ß and sclerostin on the expression of Wnt/ß-catenin signaling components at early and late stages of chondrogenic differentiation in ATDC5 cells. It appears that IL-1ß inhibits the expression of both positive and negative regulators of Wnt/ß-catenin signaling at early stages of chondrogenic differentiation, and promotes their expression in terminal calcification at late stages of chondrogenic differentiation. In addition, sclerostin counteracts the effects of IL-1ß at late stages but not at early stages. However, the present version failed to address the issues raised in my previous review. In particular, it remains unclear whether IL-1ß inhibits Wnt/ß-catenin signaling at early stages of chondrogenic differentiation, and promotes Wnt/ß-catenin signaling at late stages of chondrogenic differentiation.*

*I understand that IL-1ß may exert differential effects at early and late stages of chondrogenic differentiation. Nevertheless, IL-1ß seems to regulate the expression of both positive and negative regulators of Wnt/ß-catenin signaling. Thus, the net outcome on Wnt/ß-catenin signaling is unclear*.

**Response:** IL-1β modulates the chondrogenic differentiation and Wnt/ß-catenin signaling, but the effects differ between the early and late stages. Previous studies reported that IL-1β impaired initial chondrogenic differentiation from mesenchymal cells [1, 2]. The mechanisms may be associated with downregulation of wnt/β-catenin signaling due to negative feedback by the interaction between Wnt/β-catenin signaling and the others such as NF-κB [3], although details are unclear. When the cells were treated with IL-1β from the beginning in the present *in vitro* culture system of chondrogenic differentiation, initial chondrogenic differentiation was inhibited and wnt/β-catenin signaling was downregulated in the early stage. But once chondrogenic differentiation was progressed, IL-1β upregulated wnt/β-catenin signaling in the late stage of chondrogenic differentiation and promoted endochondral ossification. This mechanism is supported by the other studies [4, 5].

References

1. Simsa-Maziel S, Monsonego-Ornan E. Interleukin-1beta promotes proliferation and inhibits differentiation of chondrocytes through a mechanism involving down-regulation of FGFR-3 and p21. Endocrinology. 2012; 153: 2296-310.
2. Murakami S, Lefebvre V, de Crombrugghe B. Potent inhibition of the master chondrogenic factor Sox9 gene by interleukin-1 and tumor necrosis factor-alpha. J Biol Chem. 2000; 275: 3687-92.
3. Ma B, van Blitterswijk CA, Karperien M. A Wnt/β-catenin negative feedback loop inhibits interleukin-1-induced matrix metalloproteinase expression in human articular chondrocytes. Arthritis Rheum. 2012; 64:2589-2600.
4. Mumme M, Scotti C, Papadimitropoulos A, Todorov A, Hoffmann W, Bocelli-Tyndall C, et al. Interleukin-1beta modulates endochondral ossification by human adult bone marrow stromal cells. Eur Cell Mater. 2012; 24: 224-36.
5. Scotti C, Piccinini E, Takizawa H, Todorov A, Bourgine P, Papadimitropoulos A, et al. Engineering of a functional bone organ through endochondral ossification. Proc Natl Acad Sci U S A. 2013; 110: 3997-4002.

*The effect of sclerostin on IL-1ß at late stages of chondrogenic differentiation is likely indirect. Sclerostin binds to LRP5/6 to inhibit Wnt/ß-catenin signaling, and it is believed that the antagonistic effect of sclerostin on bone formation is mediated by Wnt signaling. Thus, there is no sufficient novelty in the present study.*

**Response:** IL-1β inhibits the expression of sclerostin/SOST in ATDC5 cells (Supple Fig. 2). As a possible mechanism, the stimulatory effects of IL-1β on Wnt/β-catenin signaling may be mediated by regulating interaction between sclerostin and LRP6. Suppression of sclerostin by IL-1β may upregulate LRP6 phosphorylation and downstream of Wnt/β-catenin signaling is thought to be activated. In this condition, addition of sclerostin canceled the upregulation of Wnt/β-catenin signaling.

*It is difficult to understand how the experiments were done, in particular, when the early effects were analyzed after treatment. For example, the results presented in Fig. 1C seem to be obtained after 3 weeks, but it is not clear when the cells were treated, and they represent early or late effect?*

**Response:** We apologize inaccurate description regarding treatment of cells. This point was revised in the methods section (Line 74). The cells were treated with IL-1β and/or sclerostin for 3 weeks as the early stage experiment. In the present in vitro culture system, it takes at least 7 weeks to complete whole process of chondrogenic differentiation from mesenchymal chondroprogenitor to terminal calcification. The chondrogenic differentiation initiates to form cartilage nodules through a cellular condensation process that gave rise to proliferating chondrocytes and no hypertrophic chondrocytes are present by 3 weeks. After 5 weeks, mineralized regions began to appear in the preexisting cartilage nodules as tiny spots and they propagated throughout the cartilage nodules with time. Thus, the cells at 3 weeks were included in the early stage.
